# New Appliances and Things Medical

**Published:** 1905-07-01

**Authors:** 


### NEW APPLIANCES AND THINGS MEDICAL.

[We shall be glad to receive, at our Office, 28 & 29 Southampton Street, Strand, London, W.C., from the manufacturers, specimens of all new preparations and appliances which may be brought out from time to time.]

### MOSQUERA'S BEEF MEAL.

(Mosquera-Julia Food Company, Detroit, Mich., U.S.A.)

We have received a sample of this well-known preparation. It consists essentially of powdered beef partially predigested. We believe the agent used for this purpose is the proteolytic ferment contained in pine-apple juice. We do not know exactly how far digestion has been allowed to proceed, but the substance contains, according to Chittendon, who made an analysis of it, 77 per cent. of proteid substance and 13 per cent. of fat, about half the proteids being present as peptones or albumoses. The dose of this substance is a teaspoonful three or four times a day, and it may be given in milk or added to thick soups, or spread upon bread and butter in the form of a sandwich. Beef powders are certainly the most nutritious preparations of meat available, since they consist essentially of the meat deprived of its 75 per cent. of water. We think these powders might with the greatest advantage be used much more frequently than they are.

### LAGER BEER AND STOUT.

(Allsopp's Brewery, Burton-on-Trent.)

Some years ago, when English-made Lager Beer was yet a novelty, we commented very favourably on that produced by Messrs. Allsopp, recommending especially its purity from a chemical and hygienic point of view. Many rivals have appeared in the field since then, but they have been unable to reduce the popularity of Messrs. Allsopp's Lager Beer, which holds its own as an excellent substitute for the foreign-made article, concerning the processes of production of which we cannot be so secure as in the case of our own high class manufacture. Lager Beers grow in favour in the summer, when they are undoubtedly preferable for general consumption to the heavier class of malt liquors. Messrs. Allsopp's stout may be safely recommended for invalids.

